# Genetic polymorphisms in vitamin E transport genes as determinants for risk of equine neuroaxonal dystrophy

**DOI:** 10.1111/jvim.16924

**Published:** 2023-11-08

**Authors:** Yunzhuo Ma, Sichong Peng, Callum G. Donnelly, Sharmila Ghosh, Andrew D. Miller, Kevin Woolard, Carrie J. Finno

**Affiliations:** ^1^ Department of Population Health and Reproduction School of Veterinary Medicine, University of California‐Davis Davis, California 95616 USA; ^2^ Department of Biomedical Sciences, Section of Anatomic Pathology Cornell University College of Veterinary Medicine Ithaca, New York 14853 USA; ^3^ Department of Pathology and Immunology School of Veterinary Medicine, University of California‐Davis Davis, California 95616 USA; ^4^ Present address: Eclipsebio San Diego, California 92121 USA; ^5^ Present address: Cornell University College of Veterinary Medicine Ithaca, New York 14853 USA

**Keywords:** ataxia, equine degenerative myeloencephalopathy, genetics, horse

## Abstract

**Background:**

Equine neuroaxonal dystrophy/equine degenerative myeloencephalopathy (eNAD/EDM) is an inherited neurodegenerative disorder associated with vitamin E deficiency. In humans, polymorphisms in genes involved in vitamin E uptake and distribution determines individual vitamin E requirements.

**Hypothesis/Objectives:**

Genetic polymorphisms in genes involved in vitamin E metabolism would be associated with an increased risk of eNAD/EDM in Quarter Horses (QHs).

**Animals:**

Whole‐genome sequencing: eNAD/EDM affected (n = 9, postmortem [PM]‐confirmed) and control (n = 32) QHs. Validation: eNAD/EDM affected (n = 39, 23‐PM confirmed) and control (n = 68, 7‐PM confirmed) QHs. Allele frequency (AF): Publicly available data from 504 horses across 47 breeds.

**Methods:**

Retrospective, case control study. Whole‐genome sequencing was performed and genetic variants identified within 28 vitamin E candidate genes. These variants were subsequently genotyped in the validation cohort.

**Results:**

Thirty‐nine confirmed variants in 15 vitamin E candidate genes were significantly associated with eNAD/EDM (*P* < .01). In the validation cohort, 2 intronic *CD36* variants (chr4:726485 and chr4:731082) were significantly associated with eNAD/EDM in clinical (*P* = 2.78 × 10^−4^ and *P* = 4 × 10^−4^, respectively) and PM‐confirmed cases (*P* = 6.32 × 10^−6^ and 1.04 × 10^−5^, respectively). Despite the significant association, variant AFs were low in the postmortem‐confirmed eNAD/EDM cases (0.22‐0.26). In publicly available equine genomes, AFs ranged from 0.06 to 0.1.

**Conclusions and Clinical Importance:**

Many PM‐confirmed cases of eNAD/EDM were wild‐type for the 2 intronic *CD36* SNPs, suggesting either a false positive association or genetic heterogeneity of eNAD/EDM within the QH breed.

**Abbreviations**
AVED: ataxia with vitamin E deficiency
EDM: equine degenerative myeloencephalopathy
eNAD: equine neuroaxonal dystrophy
GERP: genomic evolutionary rate profiling
IGV: integrated genome viewer
MAF: minor allele frequency
pNfH: phosphorylated neurofilament heavy
QH: Quarter Horse
SNP: single nucleotide polymorphism
TTP: tocopherol‐associated transfer protein (alpha)

## 1 INTRODUCTION

Equine neuroaxonal dystrophy/equine degenerative myeloencephalopathy (eNAD/EDM) is an inherited neurodegenerative disease associated with vitamin E deficiency during the first year of life.^1^, ^2^ Clinical signs of eNAD/EDM include symmetric ataxia (≥grade 2/5), wide‐base stance at rest, proprioceptive deficits, and decreased serum vitamin E, specifically α‐tocopherol, concentrations.^2^, ^3^, ^4^ Even after 1 year of age, serum α‐tocopherol concentrations typically remain lower in eNAD/EDM horse as compared to age‐matched healthy controls.^5^ Equine NAD/EDM can be prevented in genetically susceptible foals by supplementing dams with high doses of water‐soluble RRR‐α‐tocopherol during the last trimester of gestation, with continued supplementation in these foals through the first 2 years of life.^3^ Currently, the only way to conclusively diagnose eNAD/EDM is through postmortem histologic evaluation of the brainstem and spinal cord at necropsy.^2^ While a recently developed biomarker test for phosphorylated neurofilament heavy chain (pNfH) has demonstrated some specificity for a diagnosis of eNAD/EDM in serum, the overall sensitivity is low.^6^

In humans, ataxia with vitamin E deficiency (AVED), an inherited disease caused by deleterious variants in tocopherol transfer protein (alpha; *TTPA*), shares clinicopathologic features with eNAD/EDM.^7^, ^8^ TTP(A) is the major protein involved in transferring α‐tocopherol into liver secreted plasma lipoproteins.^9^ Equine NAD/EDM is not associated with genetic mutations in *TTPA*.^10^ However, polymorphisms in another gene involved in vitamin E uptake, distribution and metabolism could potentially modulate the risk of a horse developing eNAD/EDM. Vitamin E absorption^11^ and transport to target tissues^3^, ^12^ are not altered with eNAD/EDM. However, vitamin E metabolism is increased in Quarter Horses (QHs) with eNAD/EDM.^13^ Additionally, there is greater hepatic expression of *CYP4F2*, the major metabolizer of vitamin E, in eNAD/EDM horses compared to unaffected horses.^13^ Thus, while we elected to profile all known vitamin E candidate genes, we hypothesized that genetic polymorphisms in genes involved in vitamin E metabolism, specifically *CYP4F2*, would be associated with increased risk of eNAD/EDM.

## 2 MATERIALS AND METHODS

### 2.1 Animals—whole‐genome sequencing

All animal procedures were approved by the University of California‐Davis Institutional Animal Care and Use Committees (protocols #22427 and 22477). DNA samples for molecular work were available from a biorepository of eNAD/EDM affected and control horses, with written owner consent obtained for all postmortem sample collections of client‐owned horses. Whole‐genome sequencing was performed on n = 9 eNAD/EDM affected and n = 32 control QHs All horses for whole‐genome sequencing were QHs and QH‐related breeds (Paint, Appaloosa) and eNAD/EDM affected horses were postmortem‐confirmed, as previously described.^2^, ^14^, ^15^ All horses underwent neurologic evaluation using the modified Mayhew scale^16^ prior to study enrollment. Control horses for whole‐genome sequencing were QHs, Paints and Appaloosa horses, maintained as part of the UC Davis Teaching and Research herd at the Center for Equine Health, with ataxia scores of 0/5 and no known history of neurologic signs.

### 2.2 Whole‐genome sequencing

Genomic DNA from the 41 horses were sequenced on the Illumina HiSeq4000 at approximately 30× coverage. Whole genome sequences were deposited in the NCBI Sequence Read Archive (https://ncbi.nlm.nih.gov/subs/sra/; SUB13501748). Fastq files trimmed for quality and reads mapped to the EquCab3.0 equine reference sequence^17^ using BWA mapping program.^18^ Mapping quality was assessed using Samtools.^19^ SNP, INDEL discovery and genotyping across all samples was performed using GATK HaplotypeCaller.^20^, ^21^ To detect larger structural variants, DELLY^22^ was run on each sample and output files were merged.

### 2.3 Candidate gene evaluation

SNPSift^23^ was first used to filter the resulting vcf file created by GATK by quality, using a variant Phred threshold of 30 (*Q* ≥ 30). For both GATK and DELLY vcf files, case/control status was assigned using SNPSift CaseControl.^23^ Candidate genes involved in vitamin E absorption, transport and metabolism in humans were identified.^24^ SNPSift^23^ was then used to filter whole‐genome vcf files into candidate gene regions using EquCab3.0 coordinates for each of the 28 vitamin E candidate genes (Table 1). We included 1 kb up‐ and down‐stream from the annotated start and stop codons using the Ensembl annotation for EquCab3.0 (http://m.ensembl.org/Equus_caballus/Info/Annotation). Lastly, candidate genetic variants were filtered based on an allelic *P* value of <.01 (CC_ALL), as determined by a Fisher's Exact Test for alleles, and annotated using SNPEff.^25^ In addition to the variant callers used, raw bam files were visually inspected using Integrative Genome Viewer^26^ in the candidate gene regions for any additional structural variants that may have been missed with DELLY, including duplications, inversions and large deletions or insertions. Candidate genetic variants identified via GATK were validated in the Integrative Genome Viewer^26^ before the validation study.

**TABLE 1:** Candidate genes involved in vitamin E absorption, transport, and metabolism in humans.^24^

Gene	Protein	Equcab3.0 Coordinates	Function
*NPC1*	Niemann‐Pick type C1	chr8:41467600‐41515877	Intracellular cholesterol trafficking
*NPC2*	Niemann‐Pick type C2	chr24:19947977‐19955898	Intracellular cholesterol trafficking
*CETP*	Cholesteryl ester transfer protein	chr3:9986030‐9997310	α‐tocopherol transfer between low‐density lipoproteins
*SLC10A2*	Apical sodium‐bile acid transporter	chr17:71484203‐71503549	Uptake of bile acids
*ABCG1*	ATP‐binding cassette sub‐family G member 1	chr26:39022856‐39088436	Membrane transporter of various molecules across cellular membranes
*ABCB1*	Multidrug resistance protein 1/P‐glycoprotein 1	chr4:31862567‐32058341	Biliary secretion of α‐tocopherol
*LDLR*	LDL‐receptor	chr7:50879405‐50909076	α‐tocopherol uptake by internalization of low‐density lipoprotein
*PLTP*	Phospholipid transfer protein	chr22:35757468‐35767355	Exchange of vitamin E between lipoproteins
*MTTP*	Microsomal triglyceride transfer protein	chr3:40433746‐40487849	Vitamin E transport
*APOE*	Apolipoprotein E	chr10:15713214‐15715042	Depletion of vitamin E
*SEC14L2* (*TAP1*)	SEC14 like lipid binding 2 (Tocopherol‐associated protein 1)	chr8:8778693‐8799702	Tocopherol binding, uptake, and transport
*SEC14L3* (*TAP2*)	SEC14 like lipid binding 3 (Tocopherol‐associated protein 2)	chr8:8748318‐8758913	Tocopherol binding, uptake, and transport
*SEC14L4* (*TAP3*)	SEC14 like lipid binding 4 (Tocopherol‐associated protein 3)	chr8:8720711‐8734963	Tocopherol binding, uptake, and transport
*TTPA*	α‐tocopherol transfer protein	chr9:22442527‐22461137	α‐tocopherol retention in plasma
*NR1I2*	Pregnane X receptor (Nuclear Receptor Subfamily 1 Group I Member 2)	chr19:41597660‐41628725	Mediating gene activation by α‐tocopherol
*LPL*	Lipoprotein lipase	chr2:49312730‐49335671	α‐tocopherol transfer
*ABCA1*	ATP binding cassette transporter A1	chr25:11072753‐11204643	Cellular secretion of α‐tocopherol
*CYP3A*	P450‐cytochromes	chr13:7033680‐7591152	α‐tocopherol metabolism
*CYP4F2*	P450‐cytochromes	chr21:208056‐223547	α‐tocopherol metabolism
*AFM*	Afamin	chr3:63930257‐63950730	α‐tocopherol transport
*SLC23A1*	Sodium coupled vitamin C transporters 1	chr14:36589429‐36602402	Regeneration of vitamin E by vitamin C
*SLC23A2*	Sodium coupled vitamin C transporters 2	chr22:18515443‐18641449	Regeneration of vitamin E by vitamin C
*HP*	Haptoglobin	chr3:22546013‐22549215	Reducing oxidative activities by binding free hemoglobin
*GSTO1*	Glutathione S‐transferase omega 1	chr1:26275004‐26287327	Regenerating vitamin E by regenerating vitamin C
*GSTO2*	Glutathione S‐transferase omega 2	chr1:26246757‐26267661	Regenerating vitamin E by regenerating vitamin C
*APOA1*	Apolipoprotein A	chr7:25388642‐25390855	Major component of high‐density lipoprotein
*SCARB1*	SR‐BI scavenger receptor	chr8:26820244‐26891044	α‐tocopherol uptake and transport
*CD36*	CD36 scavenger receptor	chr4:668285‐739404	Directly or indirectly involved in vitamin E uptake

### 2.4 Animals—validation study

To validate putative genetic associations in vitamin E candidate genes, a validation study was performed with 39 eNAD/EDM affected QHs, of which 23 were postmortem confirmed, and 68 control QHs, of which 7 were postmortem confirmed. The additional n = 16 eNAD/EDM affected QHs were phenotyped as part of a previous study from a single farm, with ataxia scores ≥2.^2^ Within these 16 affected horses, n = 9 were half‐siblings by 1 stallion, n = 3 were half‐siblings by a second stallion, n = 3 were half‐siblings by a third stallion and 1 was sired by a unique fourth stallion. Control horses were either part of the previous study, with ataxia scores of 0 (n = 21),^2^ maintained as part of the UC Davis Teaching and Research herd at the Center for Equine Health, with ataxia scores of 0/5 and no known history of neurologic signs (n = 39) or client‐owned with ataxia scores of 0 (n = 8), with a total of n = 7 confirmed as unaffected via postmortem examination.

### 2.5 MassARRAY genotyping

Thirty putative genetic variants in vitamin E candidate genes were genotyped using the MassARRAY platform (Agena Bioscience, San Diego, California, USA) through Neogen Corporation (Lincoln, Nebraska, USA). Twenty‐two variants were filtered out via quality control analysis in plink^27^; 1 failed genotyping and the other 21 had minor allele frequencies below 5%. Case‐control allelic association testing using plink^27^ was then performed on the remaining 9 putative genetic variants. A Bonferroni correction was applied to account for multiple testing (*P*_Bonf_ = .006). Two analyses were performed: (a) postmortem affected cases only vs controls and (b) all cases (clinical and postmortem) vs controls.

Significantly associated SNPs were evaluated using Ensembl (https://useast.ensembl.org/Equus_caballus/Info/Index), with Genomic Evolutionary Rate Profiling (GERP) scores^28^ and minor allele frequencies (MAF) reported. A GERP score greater than 2 was considered to have higher evolutionary constraint and therefore a prioritized putative functional variant.^28^

### 2.6 Allele frequency—public database

Allele frequencies for putative genetic variants were obtained from publicly available data from 504 horses across 47 breeds.^29^ Overall allele frequencies were calculated across the 504 horses for each significant variant identified in the validation association analysis. Allele frequencies within breeds were determined for any breed group that included ≥25 individual horses (Franchese Montagne, n = 30; QH, n = 61; Shetland, n = 51; Standardbred, n = 43; Thoroughbred, n = 54 and Warmblood (including British Warmblood, German Warmblood, Hanoverian, Holsteiner, Dutch Warmblood [KWPN], Oldenbury, Trakehner, and Westphalian, n = 32)).

## 3 RESULTS

### 3.1 Whole genome association and validation study

From the whole‐genome sequencing data of 9 postmortem‐confirmed affected QHs and 32 control QHs, 43 variants were identified in 15 vitamin E candidate genes that were significantly associated with eNAD/EDM (*P* < .01; Table S1). Of these, 13 were determined to be incorrectly classified as insertions and deletions because of repeats using IGV^26^ (Table S1, red). The remaining 30 variants were genotyped in the validation cohort of horses (n = 39 eNAD/EDM and n = 68 control).

Two intronic *CD36* SNPs (chr4:726485 and chr4:731082) were significantly associated with eNAD/EDM in both clinical (Table 2; *P* = 2.78 × 10^−4^ and *P* = 4 × 10^−4^, respectively) and PM‐confirmed cases (Table 3; *P* = 6.32 × 10^−6^ and 1.04 × 10^−5^, respectively). For chr4:731082 (rs1138626727), the SNP is predicted to be intronic in 6 annotated *CD36* transcripts (Table S2) and has a GERP score of 0.29. For chr4:726485 (rs1140908279), the SNP is predicted to also be intronic in 6 annotated *CD36* transcripts (Table S2) and has a GERP score of 1.00. Minor allele frequency scores were not available in Ensembl for either of these SNPs.

**TABLE 2:** Association testing results for clinical and postmortem confirmed eNAD/EDM cases (n = 39) vs controls (n = 68).

Chr	SNP	BP	A1	F_A	F_U	A2	CHISQ	*P*	OR
4	chr4:726485	726485	G	0.15	0.02	A	13.0	3 × 10^−4^	7.94
4	chr4:731082	731082	G	0.18	0.04	A	12.5	4 × 10^−4^	5.73
26	chr26:39083023	39083023	G	0.42	0.23	A	8.6	.003	2.44
26	chr26:39074109	39074109	T	0.5	0.36	C	3.95	.05	1.78
26	chr26:39088777	39088777	A	0.43	0.54	C	2.07	.15	0.66
4	chr4:32042041	32042041	G	0.33	0.40	T	0.86	.36	0.76
1	chr1:26287108	26287108	G	0.42	0.39	C	0.21	.64	1.14
13	chr13:7046293	7046293	C	0.43	0.42	A	0.02	.90	1.04

*Note*: A Bonferroni corrected *P* value of .006 was applied and significant SNPs that passed this threshold highlighted in blue.

Abbreviations: A1, allele 1; A2, allele 2; BP, base pair; CHISQ, chi‐squared; CHR, chromosome; F_A, frequency in affected; F_U, frequency in unaffected; OR, odds ratio; SNP, single nucleotide polymorphism.

**TABLE 3:** Association testing results for postmortem‐confirmed eNAD/EDM cases only (n = 23) vs controls (n = 68).

Chr	SNP	BP	A1	F_A	F_U	A2	CHISQ	*P*	OR
4	chr4:731082	731082	G	0.26	0.04	A	20.4	6 × 10^−6^	9.24
4	chr4:726485	726485	G	0.22	0.02	A	19.4	1 × 10^−5^	12.1
26	chr26:39083023	39083023	G	0.41	0.23	A	5.64	.02	2.34
26	chr26:39074109	39074109	T	0.48	0.36	C	2.01	.16	1.63
4	chr4:32042041	32042041	G	0.30	0.40	T	1.26	.26	0.66
1	chr1:26287108	26287108	G	0.30	0.39	C	1.08	.30	0.68
26	chr26:39088777	39088777	A	0.46	0.54	C	0.90	.34	0.72
13	chr13:7046293	7046293	C	0.41	0.42	A	0.03	.87	0.94

*Note*: A Bonferroni corrected *P* value of .006 was applied and significant SNPs that passed this threshold highlighted in blue.

Abbreviations: A1, allele 1; A2, allele 2; BP, base pair; CHISQ, chi‐squared; CHR, chromosome; F_A, frequency in affected; F_U, frequency in unaffected; OR, odds ratio; SNP, single nucleotide polymorphism.

While a significant association was detected for the 2 intronic SNPs in *CD36*, many postmortem‐confirmed eNAD/EDM cases were wild type for these variants. For chr4:726485, 12/23 postmortem‐confirmed eNAD/EDM cases were wild‐type (allele frequency 0.22; Table 3, F_A). For chr4:731082, 10/23 postmortem‐confirmed eNAD/EDM cases were wild‐type (allele frequency 0.26; Table 3, F_A).

In the clinical eNAD/EDM case analysis only (ie, not solely postmortem confirmed cases), a third SNP at chr26:39083023 (rs1140829829), which is intronic in *ABCG1*, was also significantly associated with eNAD/EDM (*P* = .003; Table 3). This SNP is predicted to be intronic in 4 annotated *ABCG1* transcripts (Table S2), has a GERP score of −1.27 and the highest population MAF in Ensembl is 0.83.

### 3.2 Allele frequencies in public database

**FIGURE 1:**
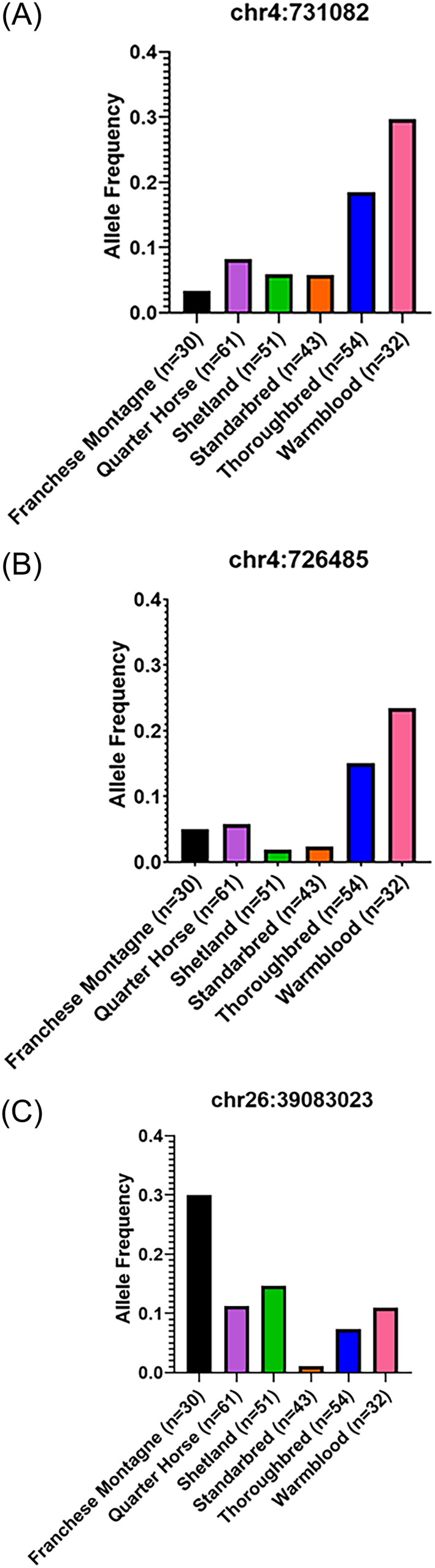
Within‐breed allele frequencies for the 3 top putative genetic variants in vitamin E candidate genes (A: chr4:731082, B: chr4:726485, and C: chr26:39083023) for equine neuroaxonal dystrophy/equine degenerative myeloencephalopathy in Quarter Horses.

The top 3 SNPs (chr4:731082, chr4:726485, and chr26:39083023) from the clinical and postmortem‐only analyses were genotyped using publicly available data from 504 horses across 47 breeds. Overall allele frequencies ranged from 0.06 to 0.1. Within‐breed allele frequencies were calculated for breeds with ≥25 horses (Figure 1; Table S3). For chr4:731082, within‐breed allele frequencies ranged from 0.03 (Franchese Montagne) to 0.30 (Warmbloods). For chr4: 726485, within‐breed allele frequencies ranged from 0.02 (Shetland and Standardbred) to 0.23 (Warmbloods). Lastly, for chr26:39083023, within‐breed allele frequencies ranged from 0.01 (Standardbred) to 0.3 (Franchese Montagne; Figure 1; Table S3).

## 4 DISCUSSION

In QHs, a postnatal vitamin E deficiency and genetic predisposition are both required to develop eNAD/EDM.^2^, ^3^ Abnormalities in vitamin E metabolism have been identified in QHs with eNAD/EDM, defined primarily by increased α‐tocopherol metabolism in both serum and urine, after administration of an oral dose of RRR‐α‐tocopherol.^13^ In that same study, there was greater hepatic expression of *CYP4F2*, the major metabolizer of vitamin E, in eNAD/EDM horses compared to unaffected horses.^13^ We therefore profiled genetic variants in genes associated with vitamin E uptake, transport and metabolism, including *CYP4F2*, in this current study. Polymorphisms in these vitamin E genes are associated with varying vitamin E levels in humans.^24^ While we did not identify any associated genetic variants in *CYP4F2*, 2 intronic *CD36* SNPs were significantly associated with eNAD/EDM in QHs in both the clinical and postmortem‐confirmed cohorts. A third SNP intronic in *ABCG1* on chr26 was significantly associated with eNAD/EDM in the clinical cohort only.

Cluster determinant 36 (CD36) plays a key role in fatty acid uptake in many tissues. This transporter is a member of the scavenger receptor family and involved in uptake of oxidized low‐density lipoproteins from the bloodstream. Alpha‐tocopherol downregulates transcription of *CD36*, both in vitro^30^ and in vivo.^31^, ^32^ In humans, a single intronic *CD36* SNP (rs1527479) is associated with plasma α‐tocopherol concentrations, and it is suggested that CD36 is involved in either intestinal absorption or tissue uptake of vitamin E.^33^ Thus, multiple lines of evidence support that CD36 participates, either directly or indirectly, in vitamin E uptake. Since intestinal absorption of eNAD horses is comparable to unaffected horses^11^ and tissue uptake into liver and CNS is also comparable after vitamin E supplementation,^3^, ^12^ variants in *CD36* are unlikely be implicated in eNAD. Additionally, the 2 eNAD/EDM associated SNPs (chr4:731082 and chr4:726485) are intronic variants in *CD36*, with GERP scores less than 2. These GERP scores do not indicate high levels of evolutionary conservation. However, there are limitations to using this approach to identify deleterious mutations, particularly in noncoding sites, since regulatory elements are not typically highly sequence conserved.^28^

While we identified a strong association between eNAD/EDM and these 2 intronic *CD36* SNPs, many postmortem‐confirmed cases did not have the alternate allele and allele frequencies were quite high for these variants in other breeds. In particular, Thoroughbreds are less likely to have eNAD/EDM^34^ and allele frequencies for these *CD36* variants on chr4 were high (0.15‐0.18) in this breed. Detailed investigation of the genotypes obtained in the validation cohort revealed that no horses in that cohort were homozygous for the minor allele (GG) for either chr4:731082 or chr4:726485. In the public dataset of 504 horses, only n = 3 were homozygous for the minor allele in each SNP (chr4:731082; German Warmblood, Holsteiner and Saddle Trotter and chr4:726485; German Warmblood, Holsteiner and Thoroughbred). The lower minor allele frequency in QHs likely led to a false association, especially since the allele frequency for both SNPs ranged from 0.06 to 0.08 in the public database of QH samples (n = 61) and the fact that many other breeds had high allele frequencies for these SNPs. Additionally, our population of QHs may contain a higher subpopulation of QHs that have a higher frequency for these genetic variants. While we aimed to match our cases and controls by subpopulation, this stratification could have led to false positive results. This highlights the importance of assessing allele frequencies across breeds after identifying putative genetic associations in order to prevent false positive results.

A third SNP on chr26 was significantly associated with eNAD/EDM in the clinical cohort only. This SNP is intronic in *ABCG1*, an ATP binding cassette protein that transports various molecules across extra‐ and intra‐cellular membranes. The transporter ABCG1 is involved in cellular vitamin E efflux and vitamin E metabolism is abnormal in *Abcg1*‐deficient mice.^35^ Due to the limited association for the *ABCG1* SNP in only the clinical eNAD/EDM analysis, and the high allele frequencies within many breeds, this *ABCG1* SNP is likely not a strong candidate variant for risk of developing eNAD/EDM.

A limitation of the current study is the biased preselection of candidate genes. While we attempted to include as many documented vitamin E candidate genes as possible, genes that warrant investigation may have been missed. Our selection of candidate genes was based on the most comprehensive review from the human literature.^24^ Additionally, although we used both an algorithm and visual inspection of reads to identify larger structural variants, errors in the reference assembly could result in missing larger genomic rearrangements within these candidate genes that could be associated with disease. Long‐range sequencing efforts are required to appropriately assemble genomes from affected and unaffected horses to identify these potential rearrangements. Despite the failure of this study to demonstrate a strong association for eNAD/EDM with any candidate variants, a genetic etiology for eNAD/EDM remains strongly supported by clinical cases in families^1^, ^36^, ^37^ and large‐scale breeding farm investigations.^2^, ^10^, ^14^, ^38^, ^39^

In conclusion, 2 intronic *CD36* SNPs were significantly associated with eNAD/EDM in QHs. However, since many PM‐confirmed cases of eNAD/EDM were wild‐type for these variants, we either identified a false positive association or genetic heterogeneity exists for eNAD/EDM within the QH breed.

## CONFLICT OF INTEREST DECLARATION

Authors declare no conflict of interest.

## OFF‐LABEL ANTIMICROBIAL DECLARATION

Authors declare no off‐label use of antimicrobials.

## INSTITUTIONAL ANIMAL CARE AND USE COMMITTEE (IACUC) OR OTHER APPROVAL DECLARATION

Approved by the University of California‐Davis IACUC (protocols #22427 and 22477).

## HUMAN ETHICS APPROVAL DECLARATION

Authors declare human ethics approval was not needed for this study.

## Supporting information


**Table S1:** Whole‐genome sequencing results at a filtered *P* value of <.01 (43 variants). Variants colored in red were incorrectly classified as insertions and deletions because of repeats, as validated using IGV,^26^ and therefore not genotyped in the validation cohort.

**Table S2:** Predicted effects for the top 3 SNPs (chr4:731082, chr4:726485, and chr26:39083023) from the clinical and postmortem‐only analyses of eNAD/EDM (https://useast.ensembl.org/Equus_caballus/Info/Index).

**Table S3:** Allele frequencies for the top 3 SNPs (chr4:731082, chr4:726485, and chr26:39083023) from the clinical and postmortem‐only analyses from publicly available data from 504 horses across 47 breeds (Table S2).
